# Room-temperature single-photon source with near-millisecond built-in memory

**DOI:** 10.1038/s41467-021-24033-8

**Published:** 2021-06-17

**Authors:** Karsten B. Dideriksen, Rebecca Schmieg, Michael Zugenmaier, Eugene S. Polzik

**Affiliations:** Niels Bohr Institute, University of Copenhagen, Copenhagen Ø, Denmark

**Keywords:** Single photons and quantum effects, Atomic and molecular interactions with photons

## Abstract

Non-classical photon sources are a crucial resource for distributed quantum networks. Photons generated from matter systems with memory capability are particularly promising, as they can be integrated into a network where each source is used on-demand. Among all kinds of solid state and atomic quantum memories, room-temperature atomic vapours are especially attractive due to their robustness and potential scalability. To-date room-temperature photon sources have been limited either in their memory time or the purity of the photonic state. Here we demonstrate a single-photon source based on room-temperature memory. Following heralded loading of the memory, a single photon is retrieved from it after a variable storage time. The single-photon character of the retrieved field is validated by the strong suppression of the two-photon component with antibunching as low as ${g}_{{\rm{RR| W = 1}}}^{(2)}=0.20\pm 0.07$. Non-classical correlations between the heralding and the retrieved photons are maintained for up to ${\tau }_{{\rm{NC}}}^{{\mathcal{R}}}=(0.68\pm 0.08)\ {\rm{ms}}$, more than two orders of magnitude longer than previously demonstrated with other room-temperature systems. Correlations sufficient for violating Bell inequalities exist for up to *τ*_BI_ = (0.15 ± 0.03) ms.

## Abstract (web summary)

Room-temperature single photon sources with memory capabilities are promising for quantum information processing, but are currently limited in their memory time or photon purity. Here, the authors report single photon emission with good antibunching from an atomic vapour cell source with 0.68 ms memory time.

## Introduction

Remarkable progress has been achieved with deterministic solid-state single-photon sources^1–7^. However, these sources require cryogenic temperatures to allow efficient photon interference^1,2,8^. Ultracold atoms, which has been another system of choice^9–13^, offer excellent performance at the expense of complexity of the experimental apparatus.

In comparison, room-temperature atomic systems have attracted a lot of attention due to their potential scalability, robustness, natural compatibility with atomic memories and favorable duty cycle^14–18^. The pioneering DLCZ proposal^19^ provided the route towards using an ensemble of atoms to combine single-photon generation and storage in the same system offering experimental simplicity, while enabling quantum information processing^20^ and quantum communication^21^ schemes. Envisioning quantum networks on a continental scale calls for single-photon sources capable of generating a photon on demand within a storage time comparable to photon time-of-flight between the parties, i.e., in the millisecond regime. When it comes to quantum storage time, the main challenge with room-temperature atomic ensembles is their thermal motion and/or collisional decoherence. Due to those limitations the DLCZ-type single-photon sources in room-temperature vapors have been up to now limited in their on-demand retrieval time to a few microseconds^22–24^. Another limitation on the performance of DLCZ-type sources has been the quantum readout noise^25^. An alternative route to a single-photon source on demand is to generate a single photon in one system, store it in another memory system and then retrieve it on demand. Towards realization of this approach in room-temperature gasses, storage of weak classical pulses limited to a few microseconds^26–28^ and reaching a few milliseconds^29^ has been demonstrated. Although the storage of external classical light pulses on the time scale of a second^30^ has recently been reported, its applicability to single-photon communication remains to be demonstrated.

Here we demonstrate an ensemble-based, deterministic room-temperature single-photon source exhibiting clear antibunching and a non-classical memory of 0.68(8) ms, two orders of magnitude longer than previously demonstrated with other room-temperature systems^22,23^. This has been achieved by combining three main ingredients, the principle of motional averaging^25,31^, a spin-protecting coating on the walls of the atomic vapor cell^32^, and the use of a Raman transition at the “magic detuning” for writing and retrieving the single photon.

## Results

### Experimental principle

**Fig. 1:**
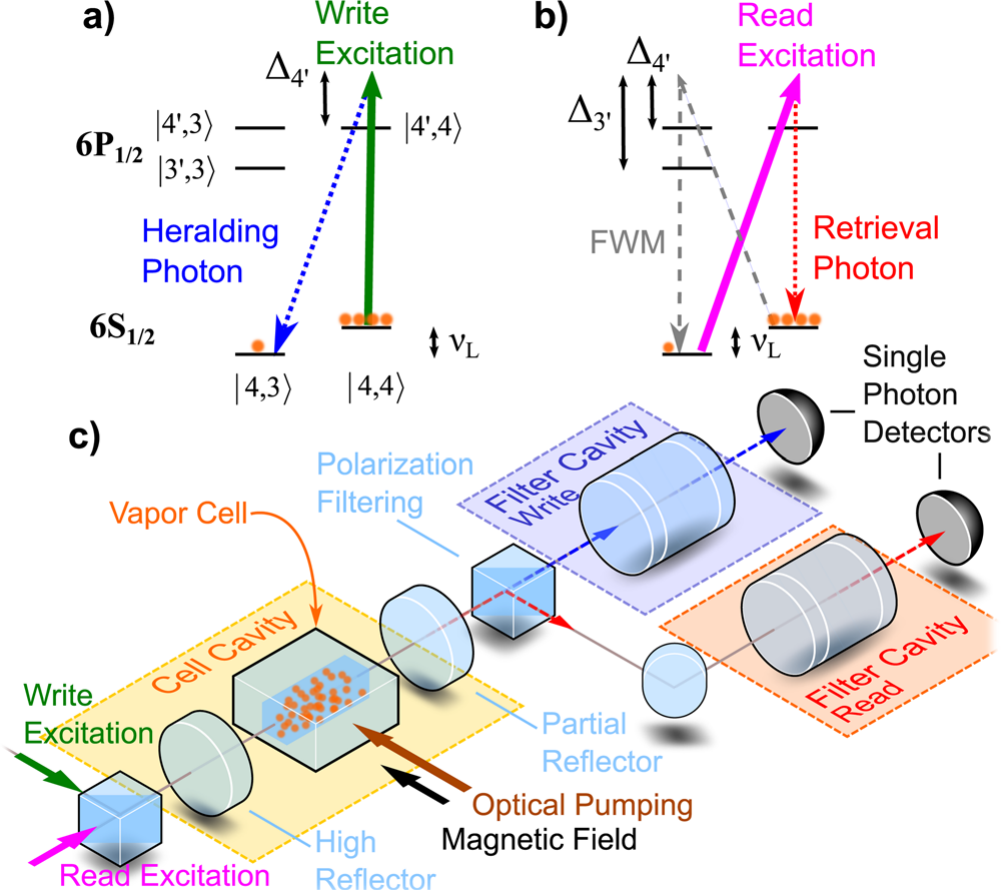
Experimental setup and excitation schemes. **a** Write excitation scheme: *π*-polarized, far-detuned excitation light creates an atomic excitation via a Raman scattering process. Only relevant atomic levels shown. **b** Read excitation scheme: *σ*-polarized light used to retrieve stored excitation via Raman scattering and scattering desired deterministic single photon. Excess four-wave-mixing (FWM) noise is suppressed by choosing ${{{\Delta }}}_{4^{\prime} }=924$ MHz . **c** Schematic of simplified experimental setup including paths for write and read scattered photons through polarization and spectral filtering.

We create a single collective excitation of the atomic ensemble when a heralding “write” photon generated via spontaneous Raman scattering is detected (Fig. 1a). Usually, the Gaussian transverse profile of the excitation beam leads to in-homogeneous coupling to the atoms, and therefore the detection of the heralding photon corresponds only to a snap-shot of the atomic positions. Consecutive atomic motion changes these positions and renders the subsequent retrieval of the photon inefficient. To remedy the effect of atomic motion, we use motional averaging to project the ensemble onto the symmetric Dicke state with equal weights for all atoms^33^. This is achieved by narrowband filter cavities (Fig. 1c), extending the duration of the detection mode of the heralding photon beyond the transverse transit time of atoms through the cell channel. As the atoms travel through the beam, the random delay from the filter cavity leads via motional averaging to washing out the which-path information of the photon, and thus equalizing the contribution of all atoms to the single collective excitation. The antirelaxation coating of the walls preserves the spin state of the atoms for thousands of collisions, extending the lifetime of the symmetric collective excitation. Four-wave-mixing (FWM) noise has been identified to be the main limitation for room-temperature vapor schemes^25,34,35^. Several strategies have been pursued to suppress this noise including ladder schemes^17,18^, cavity engineering^36^, absorption^37^, or Raman absorption^38^. An idea that is also suitable for Raman transitions between Zeeman levels is to use polarization selection rules^35,39^. However, as a result of interfering excitation paths, this generally suppresses the Raman transitions as shown in ref. ^40^. Here, we turn this effect to our advantage, exploiting a magic detuning (Fig. 1b) to suppress only the undesired FWM transition by the destructive interference of Raman amplitudes via coupling to different excited states (see “Methods” section).

### Experimental setup

In the experiment the atomic ensemble is a thermal cesium vapor with a cross-section of 300 μm × 300 μm and a length of 10 mm. The small cell cross-section enables fast motional averaging and high intensity of the laser pulses. The *N* atoms of the atomic ensemble are initially optically pumped into the coherent spin state $\left|g\right\rangle =\left|{g}_{1}...{g}_{{\rm{N}}}\right\rangle$, where $\left|{g}_{{\rm{i}}}\right\rangle =\left|F=4,{m}_{{\rm{F}}}=4\right\rangle$. Typically, we achieve an atomic polarization of 99.2%. Afterwards, a collective excitation is written into the atomic ensemble with low probability using a far-detuned, *π*-polarized write pulse (Fig. 1a). Upon detection of a scattered heralding single photon, the atomic ensemble is ideally projected onto a long-lived, symmetric Dicke state $\left|s\right\rangle =\mathop{\sum }\nolimits_{i = 1}^{N}\frac{1}{\sqrt{N}}\left|{g}_{1}...{s}_{{\rm{i}}}...{g}_{{\rm{N}}}\right\rangle$ acting as the memory storage state with $\left|{s}_{{\rm{i}}}\right\rangle =\left|4,3\right\rangle$. This choice of Zeeman configuration is beneficial for long storage time^30^. The cell is subject to a magnetic field providing a frequency splitting of *ν*_L_ = 2.4 MHz between the relevant Zeeman levels. To enhance light-atom interaction, the vapor cell is placed in an asymmetric linear cavity (Fig. 1c) with finesse ${\mathcal{F}}\approx 13$, a compromise between interaction enhancement and photon output coupling. The orthogonal polarization of the heralding photon with respect to the excitation light, and the relative detuning by one Larmor frequency *ν*_L_ facilitates filtering of the heralding photon from the 10^7^ excitation photons in the same spatial mode by polarization filtering optics, and subsequent spectral filtering with narrowband filter cavities (Fig. 1c). The filter cavities simultaneously serve the purpose of motional averaging by adding random delays to the scattered photons^31^, selecting the symmetric Dicke state. Combining motional averaging and narrow-band filtering enables a heralding probability of 82% in the symmetric mode (see Supplementary Note 2).

After a variable delay *τ*_D_, a *σ*-polarized read pulse retrieves the stored collective excitation coherently in form of a deterministic “retrieval” single photon (Fig. 1b). The filtered heralding and retrieval single photons are detected using two superconducting nanowire single-photon detectors (SNSPD).

The excitation light and single photons propagate along the cell axis orthogonal to the quantization axis defined by the optical pumping and magnetic field (Fig. 1c). The write excitation light in *π*-polarization (solid green arrow in Fig. 1a) generates the write photon (dashed blue) in the polarization mode orthogonal to the quantization axis. In the read process the latter polarization mode is used by the linearly-polarized excitation light. Beside the desired *σ*_+_-polarized component (solid pink arrow in Fig. 1b) this linearly-polarized mode contains also the undesired *σ*_−_-polarized component (dashed diagonal line in Fig. 1b), which drives the FWM process contaminating the stored excitation and adds noise to the retrieved light. By choosing the magic detuning ${{{\Delta }}}_{4^{\prime} }=924$ MHz for the read we effectively suppress this FWM process that turns out to be critical for the purity of the generated state.

**Fig. 2:**
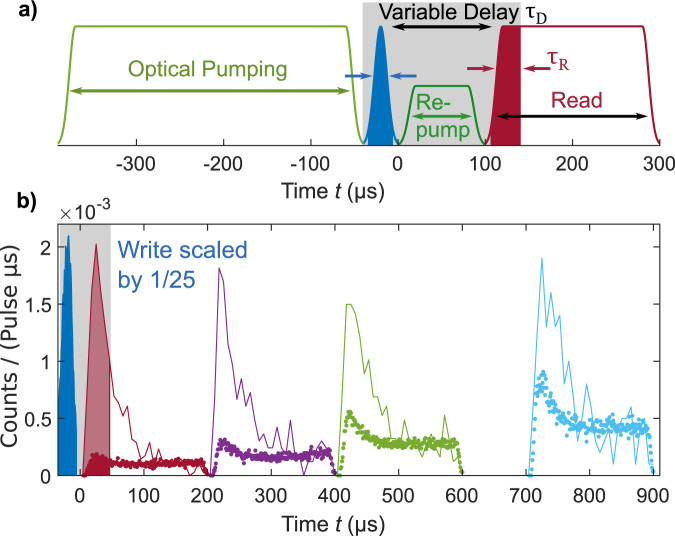
Experimental pulse sequence and temporal shape of detection events. **a** Illustration of smoothened write, read and optical pumping pulses, variable delay between write and read, and optional repump for delayed readout. The solid areas represent integration windows used in the analysis. **b** Blue area—detected counts during heralding write pulses (31 μs, scaled 1/25). Solid curves—detected counts during read pulses (200 μs, delayed 10 to 710 μs) conditioned on the heralding write count (averaged over 7 μs bins). Dotted curves—the read noise level in the absence of write pulse (1 μs binning). Source data are provided as a Source Data file.

The experimental sequence consists of two main parts, an optical pumping and locking window for all cavities, and a window containing the experimental write-read pulse sequence. The latter (Fig. 2a) contains a 350 μs optical pumping pulse for state initialization, a 40 μs write pulse, a variable delay *τ*_D_, and a 200 μs read pulse. All pulses are turned on and off smoothly to prevent high frequency harmonics which can falsely excite the memory state. The sequence of write, read and optical pumping pulses is repeated up to 75 times, depending on *τ*_D_ before re-locking of the cavities becomes necessary. For delay times of 100 μs and longer, an additional repump pulse is used to counteract birefringence effects in the cell cavity due to the atomic polarization decay.

The retrieved light follows an exponentially decaying temporal envelope (Fig. 2b). One component of the atomic noise follows the same envelope while the second component grows linearly during the delay and the pulse duration (the noise origin is discussed below). Hence, the signal-to-noise ratio (SNR) depends on the time window chosen in post-processing. We find that truncating the write window to 31 μs out of the 40 μs pulse duration and the read window at *τ*_R_ = 40 μs offers a good trade-off between SNR and retrieval efficiency, see Supplementary Note 4.

The temporal shape of the read noise validates that FWM noise is suppressed below other sources of read noise. Indeed, dominating FWM noise would mean that the read noise grows during the readout^41^, as was observed in ref. ^25^. The exponential decay in the read noise shows that the FWM noise is negligible.

### Photon correlations

The conditional generation of a single excitation in the atomic memory is characterized by the non-classical cross-correlations between the single photons scattered during the write and consecutive read pulses. The relevant 2nd-order cross-correlation is given by ${g}_{{\rm{WR}}}^{(2)}$ = 〈*n*_W_*n*_R_〉/(〈*n*_W_〉〈*n*_R_〉) where *n*_W_(*n*_R_) is the number of detection events during the write (read) process.

A long temporal shape of the retrieved light in the tens of microseconds range provides an advantage for the characterization of the photon source. Under those conditions the SNSPD, for which the dead time is less than 50 ns, works as a photon-number-resolving detector. This capability allows for accurate accounting of multiphoton events, which would otherwise compromise the accuracy of the measurement of correlation functions.

In the absence of losses and extra noise the joint state of the write photon and the memory is of the two-mode squeezer type:

1 $$\left|{{{\Psi }}}_{{\rm{uncond.}}}\right\rangle =\sqrt{1-{p}_{0}}\left({\left|0\right\rangle }_{{\rm{W}}}{\left|0\right\rangle }_{{\rm{A}}}+\sqrt{{p}_{0}}{\left|1\right\rangle }_{{\rm{W}}}{\left|1\right\rangle }_{{\rm{A}}}+{p}_{0}{\left|2\right\rangle }_{{\rm{W}}}{\left|2\right\rangle }_{{\rm{A}}}+{\mathcal{O}}\left({p}_{0}^{3/2}\right)\right),$$

where *p*_0_ is the probability of creating one or more excited pairs. ${\left|n\right\rangle }_{{\rm{W}}}$ (${\left|n\right\rangle }_{{\rm{A}}}$) refers to *n* excitations of the write scattered field (symmetric excitations in the atomic ensemble). Thus, the multiple-pair excitation probability *p*_0_ has to be kept low enough to avoid falsely heralding the single-pair state due to limited detection of the heralding field (e.g., propagation losses). We can directly relate the excitation probability to the mean number of excitations 〈*n*_exc_〉 via *p*_0_ = 〈*n*_exc_〉/(1 + 〈*n*_exc_〉). For low number of excitations and neglecting noise, this gives *p*_0_ ≈ 〈*n*_W_〉/*η*_X_, which is the mean number of detected write counts 〈*n*_W_〉 scaled with the write detection efficiency *η*_X_ = 2.9% that includes the outcoupling from the cell cavity, propagation efficiencies through the filter setup and the quantum efficiency of the detector, see Supplementary Note 3.

**Fig. 3:**
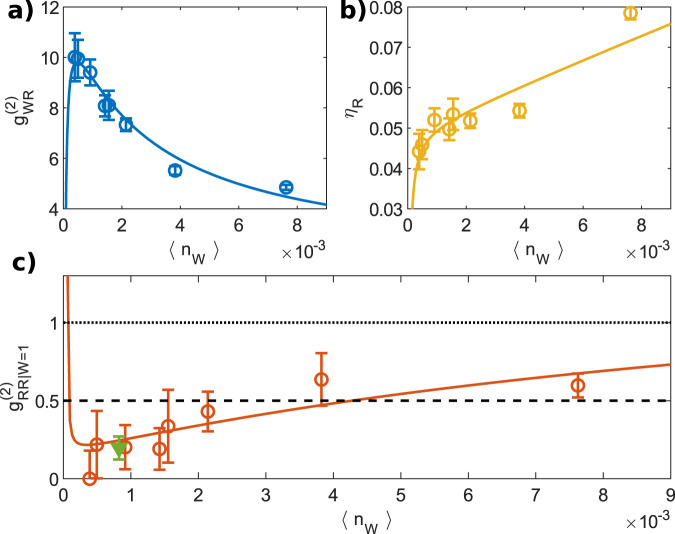
Photon correlations and retrieval efficiency. **a** 2nd-order cross-correlation function ${g}_{{\rm{WR}}}^{(2)}$, **b** retrieval efficiency *η*_R_ and **c** 2nd-order conditional auto-correlation function ${g}_{{\rm{RR| W = 1}}}^{(2)}$ of the retrieved light field. ${g}_{{\rm{RR| W = 1}}}^{(2)}$ = 1 (dotted line) is the classical limit and ${g}_{{\rm{RR| W = 1}}}^{(2)}$ = 0.5 (dashed line) is the two-photon Fock state auto-correlation value. All functions are plotted against the mean number of detected write counts $\left\langle {n}_{{\rm{W}}}\right\rangle$. Shown are measured data (circles) and the model (full lines). To improve the experimental uncertainty on ${g}_{{\rm{RR| W = 1}}}^{(2)}$ we combine points for $\left\langle {n}_{{\rm{W}}}\right\rangle \,<\,2\,\times\,1{0}^{-3}$ (green triangle). Error bars represent one standard deviation. Source data are provided as a Source Data file.

With the decreased write pulse energy and thus 〈*n*_W_〉, ${g}_{{\rm{WR}}}^{(2)}$ grows as seen in Fig. 3a as a low multipair generation probability is crucial for a high cross-correlation between the write and read fields^21^. When *n*_W_ would be decreased even further, we expect the detection events of the write field to be dominated by background noise limiting the correlations. The high value of ${g}_{{\rm{WR}}}^{(2)}$ ≈ 10 obtained for low *n*_W_ testifies to the high heralding efficiency of the excitation storage in the memory and its consecutive readout.

The retrieval efficiency ${\eta }_{{\rm{R}}}=\left\langle {n}_{{\rm{R| W = 1}}}\right\rangle -\left\langle {n}_{{\rm{noise}}}\right\rangle$ is defined as the difference between the mean number of counts conditioned on a single heralding write count, and the mean number of detected noise read counts in the absence of a write pulse (see Supplementary Note 4). As $\left\langle {n}_{{\rm{W}}}\right\rangle$ grows (Fig. 3b), *η*_R_ first grows rapidly as the write dark counts become negligible, and then continues to grow slower as the heralded state acquires an increasing contribution of multiple stored excitations.

To gain more insight, we model the system as a two-mode squeezed state with uncorrelated noise using probability generating functions (see Supplementary Note 3). The model yields the mean detected count rates, the cross-correlation, the retrieval efficiency and the conditional auto-correlation. The atomic noise contributions used in the model are found experimentally from the spectral scans of the filter cavities with and without sending a write pulse (see Supplementary Note 2). The only free fit parameters remaining in the model are the detection and intrinsic retrieval efficiencies. These are determined by simultaneously fitting to ${g}_{{\rm{WR}}}^{(2)}$, *η*_R_, and 〈*n*_R_〉. We observe good agreement of the experimental data with the fitted model as seen from Fig. 3. From the fit parameter we estimate the intrinsic retrieval efficiency, i.e., the efficiency of retrieving one excitation from the symmetric atomic mode into the cell cavity mode, to be ${\eta }_{{\rm{R}}}^{* }=(70\pm 8) \%$ for *τ*_R_ = 40 μs (see “Methods” section). We note that the propagation losses through the filters are not due to a spectral mismatch of the retrieved photon.

Next, we demonstrate that the memory indeed stores a single excitation which can be deterministically retrieved on-demand as a single photon. Towards this end, we measure the conditional auto-correlation function and verify the sub-Poissonian character of the retrieved field, for which ${g}_{{\rm{RR| W = 1}}}^{(2)}$ < 1. Figure 3c shows ${g}_{{\rm{RR| W = 1}}}^{(2)}$ as a function of $\left\langle {n}_{{\rm{W}}}\right\rangle$ which in the present case of number-resolving detection is defined as ${g}_{{\rm{RR| W}}\,=\,{\rm{1}}}^{(2)}\,=\,\langle {n}_{{\rm{R| W}}\,=\, {\rm{1}}}({n}_{{\rm{R| W}}\,=\,{\rm{1}}}-1)\rangle /{\langle {n}_{{\rm{R| W}}\,=\, {\rm{1}}}\rangle }^{2}$, where *n*_R∣W=1_ is the number of read detection events in each sequence with a preceding heralding write detection event. We observe good agreement between the experimental data and the model.

To improve the precision of ${g}_{{\rm{RR| W = 1}}}^{(2)}$ we combine datasets for $\left\langle {n}_{{\rm{W}}}\right\rangle <2\times 1{0}^{-3}$. According to the model, the read field is found to weakly depend on *p*_0_ in this range. Under those conditions, the write-read sequence has been repeated 3 × 10^7^ times. The observed heralded write probability of 〈*n*_W_〉 ~ 10^−3^ (green point in Fig. 3c) is comprised of *p*_0_ ~ 0.03 of the intrinsic write scattering probability and the write propagation efficiency of *η*_*X*_ = 0.029. The overall write-read efficiency is *η*_tot_ = *p*_0_*η*_X_*η*_Y_ ~ 5 × 10^−5^, where *η*_Y_ = 0.06 includes the intrinsic retrieval efficiency of 0.7 and the read propagation efficiency of 0.086 (see “Methods” section). The probability of the write event followed by the double retrieval event from the memory signifying the deviation from an ideal single photon storage and retrieval is ~2 × 10^−7^. The resulting ${g}_{{\rm{RR| W = 1}}}^{(2)}=0.20\pm 0.07$ is a clear indication of the single photon nature of our source. Furthermore, there is an appreciable margin of more than four standard deviations to the two-photon Fock state auto-correlation ${g}_{{\rm{n = 2,n = 2}}}^{(2)}=0.5$, which indicates good fidelity of the single-photon state.

### Delayed readout and memory time

The quantum memory capabilities of the system are mapped out by varying the delay time between write and read pulses from 10 to 1010 μs. In Fig. 2b we have included the histograms for conditional read counts and unconditional read noise for various delays. While kept in the dark, the atoms decay into the storage state primarily due to wall collisions. This leads to an increase of the read noise with increasing delay times *τ*_D_. Atomic decay compromises the readout in two ways, as atoms incoherently transferred into $\left|4,3\right\rangle$ contribute to the readout either by coupling to the easily retrievable symmetric mode or by coupling to the weakly retrieved asymmetric modes of the ensemble: 1) The high readout rate at the beginning of the read pulse originates from the symmetric mode which is efficiently read out. The retrieval of the incoherent excitation follows the same temporal shape as the desired stored excitation (Fig. 2b). These incoherent contributions to the symmetric mode constitute approximately half of the noise at the beginning of the read pulse (Fig. 2b and Supplementary Note 2). 2) Incoherent contributions to the asymmetric modes are read out only inefficiently. The accumulation of incoherent population in $\left|4,3\right\rangle$ and hence asymmetric modes leads to a count rate that slowly increases over time as the population in $\left|4,3\right\rangle$ grows.

Both noise contributions grow approximately linearly with *τ*_D_ degrading the SNR between conditional and unconditional readout.

**Fig. 4:**
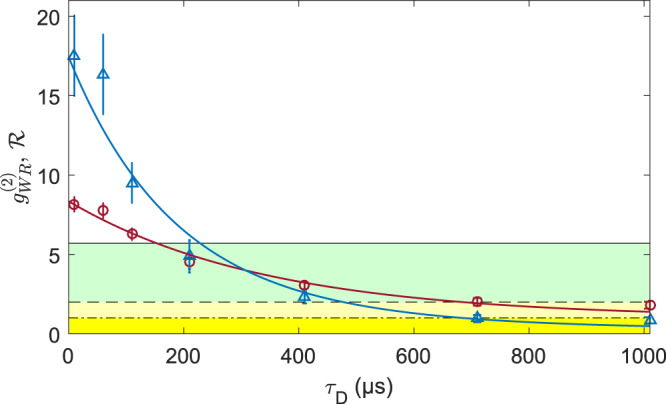
Photon correlations for delayed readout. Shown are ${g}_{{\rm{WR}}}^{(2)}$ (red) and the Cauchy-Schwarz parameter ${\mathcal{R}}$ (blue) versus various read pulse delays *τ*_D_ for an integrated read pulse duration of 40 μs together with the fit to ${g}_{{\rm{WR}}}^{(2)}$ (red line) and the resulting ${\mathcal{R}}$ (blue line). The black line marks the Bell-inequality limit ${g}_{{\rm{WR}}}^{(2)}$ ≥ 5.7 and the dashed line marks the typical non-classicallity signature ${g}_{{\rm{WR}}}^{(2)}$ > 2. The dash-dotted line is the formal non-classicallity criterion ${\mathcal{R}}$ > 1. Error bars represent one standard deviation. Source data are provided as a Source Data file.

Important characteristics of the on-demand single photon source enabled by the quantum memory are the 2nd-order cross-correlation function ${g}_{{\rm{WR}}}^{(2)}$ and the Cauchy-Schwarz parameter ${\mathcal{R}}$ = ${\left({g}_{{\rm{WR}}}^{(2)}\right)}^{2}/\left({g}_{{\rm{RR}}}^{(2)}\ {g}_{{\rm{WW}}}^{(2)}\right)$ versus delay time *τ*_D_^42^. The latter can be used to quantify the non-classicality of correlations between write and read intensities. In Fig. 4 the respective values for ${g}_{{\rm{WR}}}^{(2)}({\tau }_{{\rm{D}}})$ and ${\mathcal{R}}({\tau }_{{\rm{D}}})$ are shown, along with the exponential fit following ${g}_{{\rm{WR}}}^{(2)}({\tau }_{{\rm{D}}})$ = $B\cdot \exp (-{\rm{{\tau }}_{{{\rm{D}}}}}/{\tau }_{{\rm{M}}})+1$. ${g}_{{\rm{WW}}}^{(2)}$ is independent of *τ*_D_ and because noise dominates the unconditional readout, the dependency for ${g}_{{\rm{RR}}}^{(2)}$ is marginal which is what we observe. We therefore use averaged values for ${g}_{{\rm{WW}}}^{(2)}$ and ${g}_{{\rm{RR}}}^{(2)}$ together with the fit results of ${g}_{{\rm{WR}}}^{(2)}({\tau }_{{\rm{D}}})$ to plot the above expression for ${\mathcal{R}}({\tau }_{{\rm{D}}})$. From ${\mathcal{R}}$ we define the memory time as the time beyond which write and read light fields are no longer non-classically correlated, i.e., not fulfilling ${\mathcal{R}}$ > 1 (Fig. 4, dash-dotted line). We use ${\mathcal{R}}$ as a formal non-classicality bound instead of the typical signature ${g}_{{\rm{WR}}}^{(2)}$ > 2^21^. The corresponding memory time is ${\tau }_{{\rm{NC}}}^{{\mathcal{R}}}=(0.68\pm 0.08)\ {\rm{ms}}$. The limit for violating the Bell inequality is given by ${g}_{{\rm{WR}}}^{(2)}$≥ 5.7^43^. From the fit in Fig. 4 this holds for *τ*_BI_ = (0.15 ± 0.03) ms.

The non-classical memory time of the atomic ensemble is limited by noise from the atomic decay. However, the retrieval efficiency *η*_R_ is noise free due to its definition (see Supplementary Note 4), which allows us to determine the 1/*e*-lifetime of the collective excitation in the memory, amounting to ${\tau }_{{\eta }_{{\rm{R}}}}=0.8{9}_{-0.23}^{+0.49}\ \,\text{ms}\,$. The collective excitation lifetime is expected to be limited to half of the transverse macroscopic spin amplitude decay time, separately measured to be *T*_2_ = 2 ms (see “Methods” section) and to be dominated by spin relaxation due to wall collisions. On this time scale the atoms experience thousands of wall collisions. Hence, the long lifetime of the targeted symmetric atomic mode demonstrates the effect of the spin-protecting coating.

## Discussion

Our results demonstrate the capability to herald, store and read out a single long-lived collective atomic excitation from a room-temperature atomic vapor. We verify the single-photon nature of the retrieved light from observing strong photon antibunching. High cross-correlation and near-millisecond storage time at room temperature enable applications in quantum networks, where the platform can be immediately used as a building block for entanglement generation over up to 200 km. The feasibility of this was demonstrated in a recent proof-of-principle study verifying short-range entanglement of two warm atomic vapors after sub-microsecond storage through the DLCZ protocol^44^. The technological simplicity of our system facilitates the setup of a multitude of identical parallel systems, and thus a quantum repeater as well as simulator applications. Increasing the cell transverse dimension from the current 300 μm to several millimeters and using state-of-the-art coating^32^, we can expect to increase the storage time of single excitations to at least hundreds of milliseconds. The required higher rejection of the excitation light has been demonstrated in our laboratory^45^. Implementing a top-hat optical mode for improved filling of the cell with light^46^ will relax the spectral filtering requirements for motional averaging for a larger cell.

Besides the application as a source of narrowband single photons one can explore a larger phase space by accumulating excitations. Exciting applications may also arise from interfacing such quantum-state engineering with other platforms, such as cold atoms or mechanical oscillators.

## Methods

### Light

Excitation light pulses for write and read are derived from a narrowband home-built external cavity diode laser at 895 nm. It is locked via a beat-note lock with fixed detuning to the $F=4\to F^{\prime} =4$ transition of the *D*_1_ line of cesium. The write and read locking and excitation frequencies are derived using two AOMs.

### Vapor cell

In our experiments we use a cesium vapor cell with an interaction volume of 300 μm × 300 μm × 10 mm, coated with a spin-preserving anti-relaxation coating (alkane). The cell cavity mode has a 90 μm waist radius (1/*e*^2^ intensity) at the cell center. Using magneto-optical resonance spectroscopy^47^, the coherence time of the ground-state Zeeman levels was determined to be *T*_2_ = 2 ms for an operational temperature of 43 °C of the experiment and a Zeeman splitting of *ν*_L_ = 2.4 MHz.

### Data acquisition

To compensate for drifts in the experimental setup while acquiring measurement data, sequences with and without write, as well as sequences with varying delay *τ*_D_ are interleaved. Sequences without preceding write pulse are used to estimate noise levels in the readout.

### Optical pumping

During the locking and optical pumping window in the experimental sequence, the coherent atomic spin state is prepared using two circularly polarized pump and repump beams. The pumping is parallel to the magnetic field orientation. The repump laser is locked onto the $F=3\to F^{\prime} =2,3$ crossover transition, while the pump laser is locked on the transition $F=4\to F^{\prime} =4$. We determine the atomic polarization of atoms in the *F* = 4 manifold (typically > 99.2 %) using pulsed magneto-optical resonance spectroscopy. This high polarization is achieved by optimized beam geometry and by turning off the repump laser first, and keeping the pump laser turned on for a few microseconds longer.

### Polarization and spectral filtering

The leakage contribution is minimized using a half wave plate and a quarter wave plate after the cell cavity to optimize the polarization orientation, such that the polarization filtering using a Glan-Thompson polarizer reaches a suppression of 5 × 10^−5^. Following this polarization filtering stage, the spectral filtering consisting of two cavities for each of the detection setups, provides around 60 dB suppression for both detection setups.

### Magic detuning

FWM noise is due to read excitation light coupling to the state $\left|4,4\right\rangle$ and via a spontaneous Raman process creating excess excitations in $\left|4,3\right\rangle$. The associated Raman-Rabi coupling is given by *R* ∝ *g*Ω/Δ, where Δ is the detuning, and Ω (*g*) is the coupling strength for the excitation field (scattered field), respectively. These coupling strengths include the Clebsch–Gordan coefficients for the corresponding transitions. For the Raman transition coupled to multiple excited states *m* we need to sum over their contributions to the coupling *R* ∝ ∑_m_$g$_m_Ω_m_/Δ_m_, where Δ_m_ is the detuning from the respective state. For Raman transitions with Clebsch–Gordan coefficients of opposite signs, there will be a detuning where the above sum vanishes. For cesium atoms and light on the *D*_1_ line with a Raman transition between the states $\left|4,4\right\rangle$ and $\left|4,3\right\rangle$ via the excited states $\left|4^{\prime} ,3\right\rangle$ and $\left|3^{\prime} ,3\right\rangle$, the detuning where this transition is effectively suppressed lies outside the Doppler-broadened width. Including the motion of the atoms we can follow the derivation for motional averaging from^31^, adding the relevant excited states. This yields the expression for the coupling *R* ∝ ∑_m_*g*_m_Ω_m_*w*[(Δ_m_ + *iγ*/2)/Γ_D_] with the Faddeeva function *w*[*z*], the natural linewidth *γ* and the Doppler broadening Γ_D_. We find an optimal FWM suppression at a detuning of ${{{\Delta }}}_{4^{\prime} }=924$ MHz.

### Intrinsic retrieval efficiency

From the correlation model (see Supplementary Note 3) we find the fit parameter *η*_Y_ = (6.0 ± 0.2)%, which is the probability to have a detection event caused by retrieving one collective excitation in the symmetric atomic mode. Thus, it includes propagation losses, i.e., ${\eta }_{Y}={\eta }_{{\rm{d}}}{\eta }_{{\rm{esc}}}{\eta }_{{\rm{R}}}^{* }$. To estimate the intrinsic retrieval efficiency ${\eta }_{{\rm{R}}}^{* }$ we correct for the losses from two parts of the setup: 1) The efficiency *η*_esc_ of a photon generated inside the cell cavity escaping out through the outcoupling mirror. It is found from the single-pass transmission through the vapor cell *T*_cell_ and the reflectivity *R* of the outcoupling mirror as ${\eta }_{{\rm{esc}}}\approx {T}_{{\rm{cell}}}\left(1-R\right)/\left(1-R{T}_{{\rm{cell}}}^{2}\right)=(45\pm 2) \%$. 2) The detection efficiency of light in the retrieval photon mode after the cell cavity. We determine this using strongly attenuated laser light in the retrieval photon mode to be *η*_d_ = (19 ± 2)%.

### Uncertainty estimation

The error bars for conditional auto-correlation functions and the cross-correlation functions, as well as the retrieval efficiency are calculated using Poissonian errors. An exception is the error bar on the ${g}_{{\rm{RR| W = 1}}}^{(2)}$ = 0 point in Fig. 3c for the conditional auto-correlation, where no conditional double read detection event was recorded. In this case, we assign an error bar of ${g}_{{\rm{RR| W = 1}}}^{(2)}$ equal to the value if one conditional double read detection event had been recorded.

## Supplementary information

Supplementary Information-Room-temperature single-photon source with near-millisecond built-in memory

Peer Review File

## Data Availability

The data that support the findings of this study are available from the corresponding author upon reasonable request. Figure Source data are provided with this paper.

## Source data

Source Data
