# Supplementary material for: Room-temperature single-photon source with near-millisecond built-in memory
Source: Nat Commun. 2021 Jun 17;12:3699. doi: 10.1038/s41467-021-24033-8 (PMC8211654; doi:10.1038/s41467-021-24033-8)
Supplement: Supplementary file 1 — Supplementary Information-Room-temperature single-photon source with near-millisecond built-in memory [file 41467_2021_24033_MOESM1_ESM.pdf]

Supplementary Information – Room-temperature single-photon source with near-millisecond built-in memory

Dideriksen et al.

SUPPLEMENTARY NOTE 1 - EXPERIMENTAL SETUP

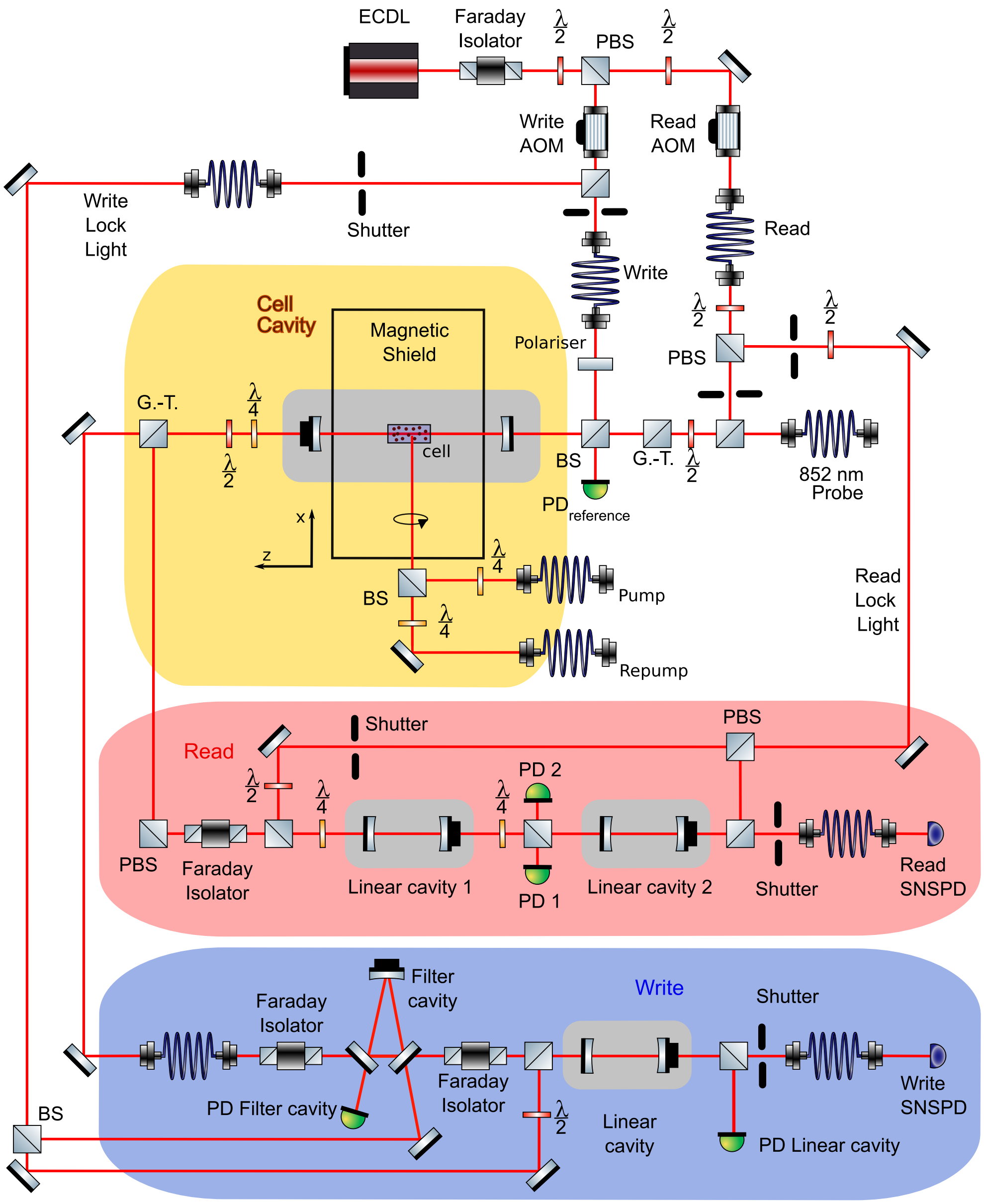

Supplementary Figure 1. **Schematic of optical setup.** Drawing of experimental setup including optical pumping, excitation and lock light paths, along with scattered photon paths through write and read filtering and detection setups. The photo detectors (PD) are used for locking. The write filtering setup consists of a triangular and a linear cavity, while for the read filtering two linear cavities are used. Further abbreviations used are Glan-Thompson polarisers (G.T.), polarising beam splitter (PBS), non-polarising beam splitter (BS), single photon detector (SNSPD), external cavity diode laser (ECDL), half-wave plate ($\lambda/2$) and quarter-wave plate ($\lambda/4$).

In Supplementary Figure 1 we present a detailed schematic of the experimental setup. It shows all paths including the excitation light paths, heralding and retrieval scattered photons and beam paths used for locking of the cavities. As can be seen in Supplementary Figure 1, the high excitation light suppression is facilitated through two consecutive cavities in each of the detection setups.

The various cavities in the setup are stabilized on resonance using different methods. The linear and the triangular cavities in the write filtering and detection setup are locked using the transmission of light modulated around the scattered write photon frequency during the locking time window of the experimental sequence. The error signal from the transmission signal is fed back onto the respective cavity's piezo-actuated mirror. For the two consecutive

linear cavities employed in the read filtering and detection setup we use dithering of the piezo-actuated mirrors to derive an error signal based on the transmission signal. The cell cavity is locked via piezo dither locking using a frequency-stabilized laser at 852 nm. Its frequency is adjusted such that we achieve simultaneous resonance of locking light (852 nm) and scattered photons (895 nm). The cell cavity linewidth is an order of magnitude broader than the Larmor frequency, thus the excitation beams are also close to resonance. Using this laser, the error signal for locking the cell cavity is generated using dithering of the piezo-actuated mirror.

SUPPLEMENTARY NOTE 2 - SPECTRUM OF WRITE AND READ SCATTERED PHOTONS

The spectrum of the write and read scattered photons is taken by detuning the frequency of the filter cavities by Δ_{FC} with respect to the photons' frequency (Supplementary Figure 2).

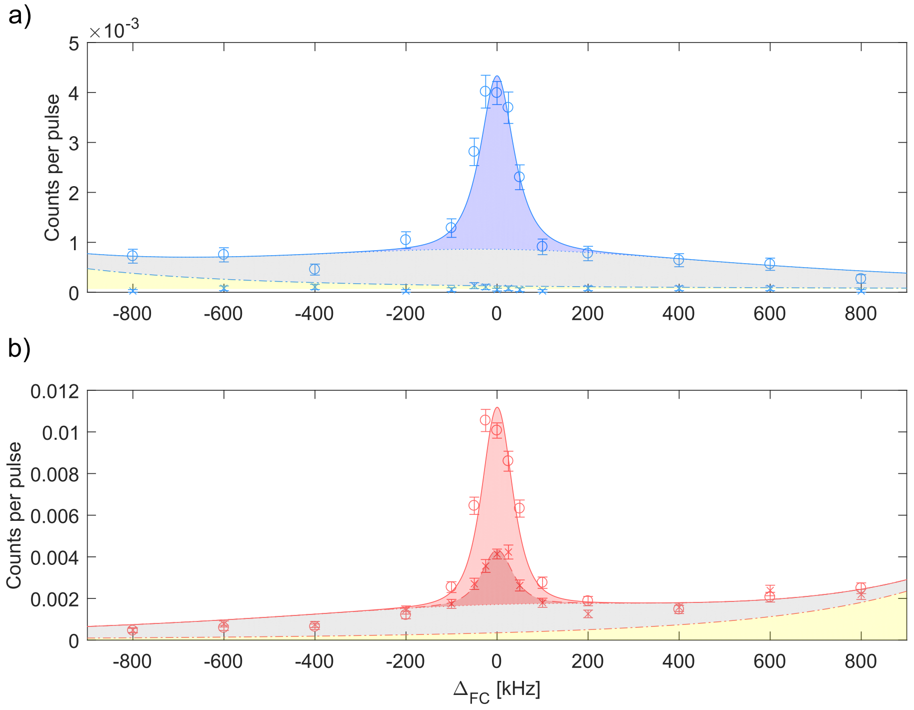

Supplementary Figure 2. **Spectrum of scattered write and read photons.** **a)** Detected unconditional Write counts per pulse. **b)** Detected unconditional Read counts per pulse with $\tau_{\mathrm{R}} = 40\,\mu\mathrm{s}$ and minimal read delay τ_{D} for different filter cavity detunings Δ_{FC} measured with write pulse (circles) and without sending a write pulse (crosses). The fits (lines) are shown with colored areas for narrowband (blue and red for write and read, respectively), broadband (gray) and leakage (yellow) contributions. Error bars represent the standard deviation assuming Poissonian distribution of count rates.

In the present excitation scheme the heralding photon (anti-Stokes photon) is scattered blue-shifted by the Larmor frequency $\nu_{\mathrm{L}} = 2.4\,\mathrm{MHz}$ compared to the write excitation light while the retrieved single photon is red-shifted by $-\nu_{\mathrm{L}}$. For the write process shown in Supplementary Figure 2a we observe a narrow peak (blue) centered at the expected heralding photon frequency. This peak results from the symmetric collective excitation while the broad peak (gray) centered at the same frequency comes from asymmetric excitations due to insufficient motional averaging. Furthermore we have a contribution from leakage (yellow) of the write excitation light centered at $\Delta_{\mathrm{FC}} = -\nu_{\mathrm{L}}$ and negligible background counts. We observe good agreement between the data (circles) and a fit (line) consisting of the sum of the narrow, broad, leakage and background contributions, which is given in the same order

$$S_{\mathrm{W}}(\Delta_{\mathrm{FC}}) = a_{\mathrm{narr}}\mathcal{L}_1(\Delta_{\mathrm{FC}},0)\mathcal{L}_2(\Delta_{\mathrm{FC}},0) + a_{\mathrm{broad}}\mathcal{L}_{\mathrm{broad}}(\Delta_{\mathrm{FC}},0) + a_{\mathrm{lkg}}\mathcal{L}_1(\Delta_{\mathrm{FC}},-\nu_{\mathrm{L}})\mathcal{L}_2(\Delta_{\mathrm{FC}},-\nu_{\mathrm{L}}) + a_{\mathrm{bg}} \tag{1}$$

where $\mathcal{L}_i(\Delta_{\mathrm{FC}},\Delta_0)$ is a Lorentzian centered at Δ_0 with unity peak value and i indexes the linewidth of the according filter cavities (1, 2) or the Lorentzian lineshape expected from the asymmetric excitations [1] (labelled "broad") and a_j is the amplitude of the respective contribution. The filter cavities are optimized for high transmission of the scattered

photons, therefore the measured width of the narrow peak is mainly limited by the width of our filter cavities. We can determine the write efficiency η_{W}, that is the probability to create a symmetric excitation conditioned on the detection of a scattered photon, as the ratio $a_{\mathrm{narr}}/(a_{\mathrm{narr}}+a_{\mathrm{broad}})$ resulting in $\eta_{\mathrm{W}} = (82\pm1)\%$. This value is improved by 20% compared to previously reported [2] thanks to an increased excitation beam waist in the cell.

For the read process shown in Supplementary Figure 2b we observe the unconditional retrieval counts (circles) above the noise level (crosses) which is measured in the absence of a preceding write pulse. Besides the desired narrowband retrieval of the stored excitation (light red) we observe a narrowband noise contribution (dark red), a broadband contribution (gray), leakage from read excitation (yellow) that is here centered at $+\nu_{\mathrm{L}}$ and negligible background counts. Imperfect initial pumping and decay over the pulse durations leads to excess atomic population in the symmetric and asymmetric modes of the state $|4,3\rangle$ resulting in the observed narrowband and broadband noise, respectively. We fit the expression

$$S_{\mathrm{R}}^{i}(\Delta_{\mathrm{FC}}) = b_{\mathrm{narr}}^{i}\mathcal{L}_1(\Delta_{\mathrm{FC}},0)\mathcal{L}_2(\Delta_{\mathrm{FC}},0) + b_{\mathrm{broad}}\mathcal{L}_{\mathrm{broad}}(\Delta_{\mathrm{FC}},0) + b_{\mathrm{lkg}}\mathcal{L}_1(\Delta_{\mathrm{FC}},+\nu_{\mathrm{L}})\mathcal{L}_2(\Delta_{\mathrm{FC}},+\nu_{\mathrm{L}}) + b_{\mathrm{bg}} \quad (2)$$

simultaneous to the two data sets $i \in \{\mathrm{W, NW}\}$, i.e. with and without preceding write pulse. Only the narrowband amplitudes b_{narr}^{i} are specific to the data sets and the rest are common fit parameters.

SUPPLEMENTARY NOTE 3 - MODELLING CORRELATIONS

In order to establish analytical expressions for the photon correlations in our experiment in the presence of noise, we describe our system in the framework of probability generating functions. The probability generating function $G_X(s)$ of a process X with outcomes x is defined as

$$G_X(s) = \mathbb{E}(s^x) = \sum_{x=0}^{\infty} \mathbb{P}(X=x)s^x. \quad (3)$$

We can derive the k'th factorial moment of the distribution

$$\mathbb{E}\{X(X-1)...(X-k+1)\} = \left.\frac{d^k}{ds^k}G_X(s)\right|_{s=1} \quad (4)$$

and express correlations as

$$G_{X,Y}(s,t) = \mathbb{E}(s^x t^y) = \sum_{x=0}^{\infty}\sum_{y=0}^{\infty} \mathbb{P}(X=x, Y=y)s^x t^y. \quad (5)$$

The system is modelled by a two-mode squeezed state which is described by a thermal distribution of photons pairs in write (X) and read (Y) with the probability generating function

$$G_{X,Y}(s,t) = \frac{1}{1+\mu(1-st)} \quad (6)$$

where μ is the mean number of excitations. Then we add noise as independent processes for write ($W = X + A$) and read ($R = Y + B$) such that

$$G_{W,R}(s,t) = G_{X,Y}(s,t)G_A(s)G_B(t) \quad (7)$$

where the noise has mean numbers λ_A and λ_B respectively. Note, that these are the detected mean numbers. The actual form of G_A, G_B is irrelevant for the derivation where only the mean values and 2nd-order correlations are needed. The limited detection efficiency during write η_X and read η_Y is accounted for by substituting $s \to 1 + \eta_X(s-1)$ and similarly for t. The detection efficiency includes all losses from generation of a photon to a detection event. For the read step this includes the intrinsic retrieval efficiency, that is the probability to generate a photon conditioned on a single collective symmetric excitation.

The probability generating functions yield an expression for the cross-correlation

$$g_{WR}^{(2)} = \frac{\mathbb{E}(WR)}{\mathbb{E}(W)\mathbb{E}(R)} = 1 + \frac{\eta_X\eta_Y(\mu^2+\mu)}{\eta_X\eta_Y\mu^2 + \mu(\eta_Y\lambda_A + \eta_X\lambda_B) + \lambda_A\lambda_B}. \quad (8)$$

The conditional auto-correlation is found by first introducing the generator for the conditional read

$$G_{R|W=1}(t) = \frac{\frac{d}{ds}G_{W,R}(s,t)\big|_{s=0}}{\frac{d}{ds}G_{W,R}(s,t)\big|_{s=0,t=1}} \tag{9}$$

and recalling the definition of the 2nd-order autocorrelation function

$$g^{(2)}_{RR|W=1} = \frac{\mathbb{E}(R(R-1))}{\mathbb{E}(R)^2} = \frac{\eta_Y^2\tilde{\mu}^2 g^{(2)}_{YY|W=1} + \lambda_B^2 g^{(2)}_{BB} + 2\eta_Y\tilde{\mu}\lambda_B}{\eta_Y^2\tilde{\mu}^2 + \lambda_B^2 + 2\eta_Y\tilde{\mu}\lambda_B} \tag{10}$$

where we have introduced the conditional mean excitation number $\tilde{\mu} = \mathbb{E}(Y|W=1)$, the auto-correlation of the read noise $g^{(2)}_{BB}$ and used

$$g^{(2)}_{YY|W=1}(\mu,\eta_X,\lambda_A) = \frac{-(2(\eta_X-1)(\lambda_A+\eta_X\mu+\lambda_A\eta_X\mu)(\lambda_A+2\eta_X-\lambda_A\eta_X+3\eta_X\mu-\eta_X^2\mu+\lambda_A\eta_X\mu-\lambda_A\eta_X^2\mu))}{(\lambda_A+\eta_X-\lambda_A\eta_X+2\eta_X\mu-\eta_X^2\mu+\lambda_A\eta_X\mu-\lambda_A\eta_X^2\mu)^2}. \tag{11}$$

Furthermore, we can calculate the retrieval efficiency $\eta_R = \eta_Y\tilde{\mu}$ and the mean detected photon number in the read process $\langle n_{\mathrm{R}}\rangle = \mathbb{E}(R)$.

The above expressions depend in total on five parameters that are determined via calibration measurements. To complete the model for different write excitation powers, we need to add a sixth parameter, that describes how the noise during the write excitation pulse λ_A changes with the write power.

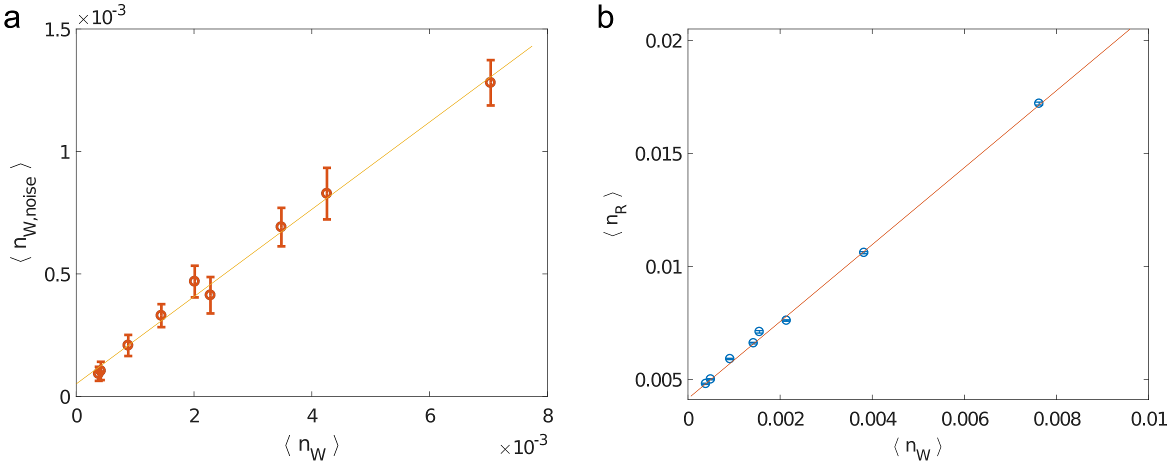

Supplementary Figure 3. **Write noise and read counts.** **a)** Mean number of noise counts per pulse $\langle n_{\mathrm{W,noise}}\rangle$ measured during the write detection window (red circles) plotted versus the mean number of write counts $\langle n_W\rangle$ with Poissonian standard deviation. Linear fit shown as yellow line. **b)** Mean number of counts per pulse $\langle n_R\rangle$ measured during the read detection window (blue circles) plotted versus the mean number of write counts. Error bars represent the standard deviation assuming Poissonian distribution of $\langle n_R\rangle$. The fitted model is shown as the red line.

We determine the write noise from the spectral analysis (Supplementary Figure 2) as the on-resonance value when subtracting the narrowband peak. We observe, that the write noise scales linearly with the mean number of write counts (Supplementary Figure 3 a). Fitting yields the offset background noise level and the slope that are then used as input parameters for the model. We observe that the read noise level λ_B and the auto-correlation of this noise $g^{(2)}_{BB}$ are constant in the write power and determine their values by averaging the measurement results taken without a preceding write excitation pulse. This leaves the two detection efficiencies as free parameters.

Those parameters are determined by simultaneously fitting $g^{(2)}_{\mathrm{WR}}$, η_{R} and $\langle n_{\mathrm{R}}\rangle$. We observe good agreement of the experimental data with the fitted model shown as the curves in the respective figure in the main text and in the supplementary Supplementary Figure 3**b**.

We obtain the the best fit values for the detection efficiencies of $\eta_X = (2.9 \pm 0.1)\%$ and $\eta_Y = (6.0 \pm 0.2)\%$.

SUPPLEMENTARY NOTE 4 - RETRIEVAL EFFICIENCY VS. DELAY

The retrieval efficiency η_R determines how well we can retrieve a stored excitation conditioned on detection of a preceding heralding write single photon, and is defined as

$$\eta_\mathrm{R} \quad = \quad \langle n_{\mathrm{R|W=1}} \rangle - \langle n_\mathrm{noise} \rangle \tag{12}$$

where $\langle n_{\mathrm{R|W=1}} \rangle$ is the mean number of read detection events conditioned on a single preceding write heralding click, and $\langle n_\mathrm{noise} \rangle$ is the mean number of read detection events obtained without write pulses. In this way, the retrieval efficiency is intrinsically corrected for noise.

In our experiment, the total read duration is 200 µs, while for the analysis we choose to integrate only over the first 40 µs. The reason is, that there is a trade-off between signal-to-noise ratio and retrieval efficiency. From Figure 2b in the main text it is apparent, that the SNR decreases for longer read pulse integration window τ_R. At the same time however, the retrieval efficiency grows with τ_R, as can be seen in Supplementary Figure 4. Note that the data points in Supplementary Figure 4 are not statistically independent since only the analysis parameter τ_R is varied.

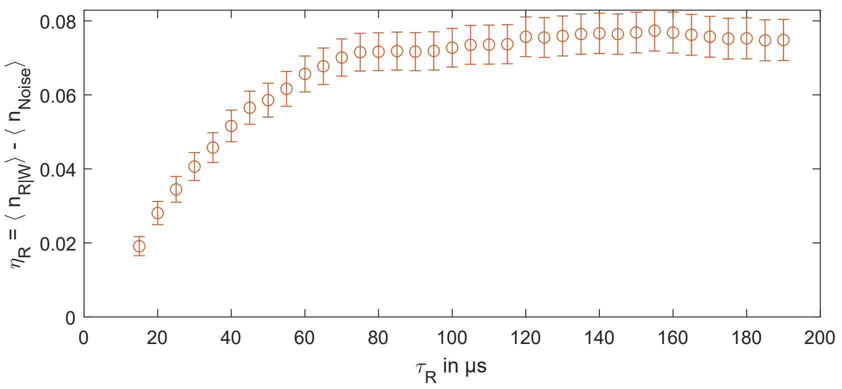

Supplementary Figure 4. **Retrieval efficiency η_R vs. integration time τ_R.** Error bars represent the standard deviation assuming Poissonian distribution of count rates.

By choosing $\tau_\mathrm{R} = 40\,\mu\mathrm{s}$, we achieve good SNR, while still maintaining high retrieval efficiency. Furthermore, the estimated intrinsic retrieval efficiency $\eta^*_\mathrm{R} = (70 \pm 8)\%$ for τ_R =40 µs indicates that we observe close to 100% of the full read pulse.

Since the retrieval efficiency is corrected for noise by definition, it can be used to determine the lifetime of the collective excitation. To this end, the retrieval efficiency is determined for various delays τ_D and the resulting values are fit to an exponential fit model:

$$\eta_\mathrm{R}(\tau_\mathrm{D}) \quad = \quad B \cdot \exp\left(-\tau_\mathrm{D}/\tau_{\eta_\mathrm{R}}\right). \tag{13}$$

From the resulting fit in Supplementary Figure 5, the memory time for the retrieval efficiency is determined as the time, during which the retrieval efficiency is reduced to its $1/e$-value, $\tau_{\eta_\mathrm{R}} = 0.89^{+0.49}_{-0.23}$ ms.

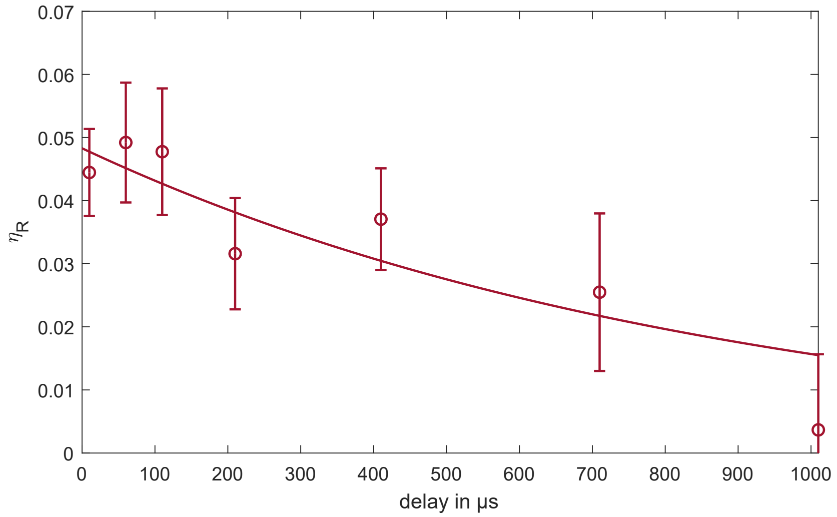

Supplementary Figure 5. **Retrieval efficiency η_R vs. delay time τ_D.** Shown is the retrieval efficiency for various delay times and a fixed readout integration duration of $\tau_R = 40\,\mu s$. Error bars on the retrieval efficiency represent the standard deviation assuming Poissonian distribution of count rates. An exponential function is fit to obtain the intrinsic memory time.

[1] J. Borregaard, M. Zugenmaier, J. M. Petersen, H. Shen, G. Vasilakis, K. Jensen, E. S. Polzik, and A. S. Sørensen, Nat. Commun. **7**, 11356 (2016).
[2] M. Zugenmaier, K. B. Dideriksen, A. S. Sørensen, B. Albrecht, and E. S. Polzik, Commun. Phys. **1**, 76 (2018).
