# Supplementary material for: Room-temperature single-photon source with near-millisecond built-in memory
Source: Nat Commun. 2021 Jun 17;12:3699. doi: 10.1038/s41467-021-24033-8 (PMC8211654; doi:10.1038/s41467-021-24033-8)
Supplement: Supplementary file 2 — Peer Review File [file 41467_2021_24033_MOESM2_ESM.pdf]

Editorial Note: This manuscript has been previously reviewed at another journal that is not operating a transparent peer review scheme. This document only contains reviewer comments and rebuttal letters for versions considered at *Nature Communications*.

Reviewers' Comments:

Reviewer #1:
Remarks to the Author:
The authors have addressed my previous comments in satisfactory way and revised their manuscript accordingly. I thus recommend its publication in the current form.
